# Identification of a Two-Gene Biomarker Correlated with Sensitivity to Combined PARP7 Inhibition and AHR Activation in Cancer Cells

**DOI:** 10.1158/2767-9764.CRC-25-0173

**Published:** 2026-01-02

**Authors:** Xuxu Gou, Huadong Chen, Morgan E. Diolaiti, Alan Ashworth

**Affiliations:** Helen Diller Family Comprehensive Cancer Center, University of California, San Francisco, San Francisco, California.

## Abstract

PARP7 inhibitors (PARP7i) and aryl hydrocarbon receptor agonists (AHRa) can synergistically suppress growth of some cancer cell lines but not others. In this study, we derived a two-gene transcriptional score to predict the synergistic response and characterized the molecular, immune, and prognostic features of this biomarker. Cancer cells were categorized into synergistic and nonsynergistic cell lines based on synergy scores that we previously reported. Published RNA sequencing data were used to establish a biomarker for PARP7i and AHRa synergistic response that was comprised of the expression of the two most differentially expressed immune-related genes (*CCL22* and *TNFSF10*). This biomarker was successfully validated in an independent set of cell lines. An integrated multiomics analysis revealed that synergistic lines had increased expression of immune-related gene sets and low expression of epithelial–mesenchymal transition–associated genes and co-expression network analysis identified transcription factors associated with synergy. In The Cancer Genome Atlas patients with breast and prostate cancers, biomarker genes were correlated with immune cell infiltration. Receiver operator characteristic (ROC) and hazard ratio (HR) analyses demonstrated that the biomarker score was correlated with response to anti–programmed cell death protein 1 (PD-1) therapy in a cohort of patients with kidney cancer and correlated with better overall survival in patients with cancer treated with anti–programmed cell death ligand 1 (PD-L1) and anti–cytotoxic T-lymphocyte–associated protein 4 therapies. In summary, we have identified a transcriptional biomarker that predicts cellular response to PARP7i and AHRa combination therapy, which is associated with benefits from immune checkpoint inhibitor therapies in patients with cancer.

**Significance:**

We employed a multiomic approach to identify a transcriptional biomarker that is predictive of cellular response to combined treatment with PARP7i and AHRa. The molecular, immune, and prognostic characterizations of this biomarker may provide insights into the molecular mechanisms of response and aid in stratifying patients likely to benefit from this combination therapy.

## Introduction

Poly (ADP-ribose) polymerase 7 (TIPARP/PARP7) is a member of the PARP family of enzymes known for their role in regulating immune response (1–3). Mechanistically, PARP7 catalyzes the transfer of a single ADP-ribose (ADPr) from NAD+ to protein targets, a process known as mono-ADP-ribosylation (MARylation; ref. 4). Unlike PARP1, which is primarily involved in DNA damage repair, PARP7 is a key regulator of immune signaling (1, 5). Although the pathways affected by PARP7 have not been fully elucidated, PARP7 has been shown to repress type I interferon (IFN) response by MARylating TBK1 and downstream targets (1, 5). This modification disrupts the ability of TBK1 to activate downstream signaling cascades, including the phosphorylation of IRF3 and subsequent induction of IFN-stimulated genes (1, 6).

Recent studies have highlighted the therapeutic potential of PARP7 inhibitors (PARP7i) as promising anticancer agents due to their ability to induce antitumor immunity. RBN-2397, a first-in-class PARP7i, has been shown to restore type I IFN signaling and induce an adaptive immune response (1). Combining RBN-2397 with anti–programmed cell death protein 1 (PD-1) agents resulted in complete and durable tumor regression in animal models (1). Based on these findings, RBN-2397 is currently being tested in clinical trials in combination with pembrolizumab, an anti–PD-1 immune checkpoint inhibitor (ICI), in patients with squamous cell carcinoma of the lung, aiming to restore response to PD-1/programmed cell death ligand 1 (PD-L1) inhibitors (NCT05127590).

PARP7 is a transcriptional target of the ligand-activated aryl hydrocarbon receptor (AHR) and its expression is upregulated in the presence of AHR ligands. In turn, PARP7 regulates AHR activity, localization, and stability, forming a negative feedback loop (7–9). AHR has been reported to modulate the development and function of innate and adaptive immune cells (10, 11). Additionally, the expression of PD-L1 in lung epithelial cells is induced by AHR and, in lung cancer, AHR expression and nuclear localization are positively correlated with response to the anti–PD-1 antibody pembrolizumab (12).

As PARP7 can regulate AHR signaling, leading to altered expression of genes involved in immune response, we explored the interplay between PARP7 and AHR, in the context of cancer therapy. We showed that PARP7i RBN-2397 can act synergistically with the AHR agonist (AHRa) tapinarof to inhibit the growth of many cancer cell lines, including those that are sensitive or resistant to single-agent RBN-2397 (8, 9). This synergistic response is dependent on AHR/AhR nuclear translocation and is associated with increased levels of nuclear AHR and increased transcription of AHR target genes. Although PARP7i and AHRa can inhibit growth of both androgen receptor (AR)–positive prostate cancer cells and estrogen receptor (ER)–positive breast cancer cells (9), not all responsive cell lines were hormone receptor positive. Understanding why some cell lines responded to this combination of drugs and others did not is essential for advancing the therapeutic potential of this approach. In this study, we developed an mRNA-based biomarker for sensitivity to the combined treatment of PARP7i and AHRa and demonstrated the potential of this biomarker to predict response.

## Materials and Methods

### Identification of a PARP7i and AHRa response biomarker

We previously measured the synergistic activity of PARP7i and AHRa across a panel of 47 cancer cell lines and reported their Bliss scores as quantification of synergy (9, 13). Of these cell lines, 38 had available RNA sequencing (RNA-seq) read count data (OmicsExpressionGenesExpectedCountProfile) in DepMap Public 24Q2 (https://depmap.org/portal/data_page/?tab=currentRelease; RRID: SCR_017655). For this study, we defined synergy as a Bliss score >10 and nonsynergy as <5, deviating from the conventional threshold of 0, to more reliably capture the robust synergistic response observed between PARP7i and AHRa in our growth inhibition experiments (9), while maintaining sufficient sample sizes in both categories. Using these criteria, 37 cell lines were divided into two groups: 27 synergistic (Bliss score >10) and 10 nonsynergistic lines (Bliss score <5), which were then randomized into two sets for training and validation using List Randomizer (https://www.random.org/lists/). In the training set, 13 synergistic cell lines (MDA-MB-468, NCI-N87, SK-BR-3, MCF-7, MDA-MB-231, CAL 27, SW620, JHOS-2, H1299, HCC1937, PEO4, T-47D, and Capan-1) were compared with five nonsynergistic lines (AGS, HAP-1, SW48, PC3, and MDA-MB-436). The validation set included 14 synergistic cell lines (OVCAR3, NCI-H1437, HT-29, 22Rv1, BT-474, A-375, PEO1, COV362, A549, LnCaP, DLD-1, HeLa, NCI-H1373, and MDA-MB-157), and five nonsynergistic lines (HGC-27, K-562, U-2 OS, HCC1395, and HCT 116).

RNA-seq read counts for cell lines in the training set were transformed into counts per million (CPM) values, and differentially expressed genes (DEG) were analyzed by comparing the 13 synergistic and five nonsynergistic cell lines in the training set using iDEP2.10 DESeq2 (RRID: SCR_027373; RRID: SCR_015687; refs. 14, 15). This comparison yielded a total of 236 upregulated DEGs in synergistic versus nonsynergistic lines with a fold change (FC) greater than eight and an adjusted *P* value less than 0.05. To reduce the impact of potential outliers and confounding variables, a leave-one-out (LOO) analysis was conducted. In each iteration, one cell line was excluded and the DEGs were rederived. The intersection of DEGs from the full comparison and all 18 LOO analyses yielded 98 consistently upregulated DEGs (Supplementary Table S1).

Two immune-related gene sets were obtained from InnateDB (https://www.innatedb.com/; RRID: SCR_006714): (i) Immunology Database and Analysis Portal (ImmPort; RRID: SCR_012804) and (ii) Immunome Database. Comparison of these immune-related gene lists with the 98 DEGs identified four immune-related genes that were upregulated in synergistic cells: *CCL22*, *CDH5*, *CX3CL1*, and *TNFSF10*. A Venn diagram was generated using BioVenn (RRID: SCR_026853; ref. 16). The predictive performance of each candidate gene was measured using receiver operator characteristic (ROC) curve analysis. Two genes, *CCL22* and *TNFSF10*, which showed significant AUC values, were combined to form a two-gene biomarker for PARP7i and AHRa response. The biomarker score was computed as the mean expression of these two genes. This biomarker was applied to both the training and validation sets, and group comparisons were performed using an unpaired *t* test in GraphPad Prism 10 (RRID: SCR_002798).

### ROC curve analysis

To evaluate the predictive power of the biomarker, as well as individual genes, proteins, or HALLMARK gene sets, nonparametric ROC analyses were performed using GraphPad Prism 10 (RRID: SCR_002798) to estimate AUC (17). The AUC represents the probability that a given signature, gene, protein, or HALLMARK gene set ranks a randomly selected synergistic cell line higher than a randomly selected nonsynergistic line.

The cutoff score for response, along with sensitivity and specificity, was derived from the ROC curve analysis in the training set. This cutoff was selected to maximize the likelihood ratio (LR), which quantifies how effectively the gene expression biomarker predicts synergy. The formula used to calculate LR was “sensitivity/(1 − specificity)” (18). The established cutoff was subsequently applied to the validation set to determine the sensitivity and specificity of the biomarker in the validation set. Cell lines with scores above the cutoff were predicted to be synergistically inhibited by PARP7i and AHRa whereas those with scores below the cutoff were classified as nonsynergistic.

The expression levels of proteins were downloaded from DepMap (https://depmap.org/portal/data_page/?tab=customDownloads; RRID: SCR_017655). The scores of HALLMARK_IFNα response, HALLMARK_IFNγ response, and HALLMARK_epithelial–mesenchymal transition (EMT) were calculated by single-sample gene set enrichment analysis (ssGSEA) using GenePattern (RRID: SCR_003201; ref. 19) to quantitatively assess these pathways in each cell line. The input for ssGSEA was a GCT file of global gene expression [log_2_ (CPM + 4)] of 37 cancer cell lines from DepMap Public 24Q2 (RRID: SCR_017655).

The expression levels of biomarker genes in patient samples and patient immunotherapy response were obtained from ClinicalOmicsDB (20). ROC analysis was performed using easyROC (http://biosoft.erciyes.edu.tr/app/easyROC/) and the curves were plotted using SRplot (http://www.bioinformatics.com.cn/srplot; RRID: SCR_025904).

To measure the predictive performance of immune markers for ICI response, we obtained AUCs with *P* values for CIBERSORT.ABS_T_cell_follicular_helper, EPIC_T_cell_CD4^+^, MCP.COUNTER_T_cell, and ESTIMATE_immune_score in the Choueiri Kidney Cancer Cohort from ClinicalOmicsDB (20).

### Correlation analysis

Pearson and Spearman correlation analyses were performed using GraphPad Prism 10 (RRID: SCR_002798) to compare Bliss synergy scores with *PARP7* mRNA levels, *AHR* mRNA levels, ssGSEA score of AHR pathway, or biomarker score. Bliss synergy scores of cell lines have been previously reported (9). The expression levels of *PARP7* and *AHR* mRNA in these lines were downloaded from DepMap Public 25Q2 (https://depmap.org/portal/data_page/?tab=customDownloads; RRID: SCR_017655). The score of AHR pathway was calculated by ssGSEA using GenePattern (RRID: SCR_003201; ref. 19).

To investigate the relationship of *CCL22* and *TNFSF10* mRNA expression across the 1684 cell lines in DepMap, Pearson and Spearman correlation analyses were performed using GraphPad Prism 10 (RRID: SCR_002798). mRNA expression data were downloaded from DepMap Public 25Q2 (https://depmap.org/portal/data_page/?tab=customDownloads; RRID: SCR_017655).

Correlations with a coefficient between 0.4 and 0.7 were considered moderately positive and above 0.7 were characterized as strongly positive.

### Overrepresentation analysis

To discover differentially regulated pathways associated with PARP7i and AHRa response, DEGs from 27 synergistic and 10 nonsynergistic cell lines were identified by iDEP2.10 DESeq2 (RRID: SCR_027373; RRID: SCR_015687; refs. 14, 15). Overrepresentation analysis (ORA) was then performed on 588 upregulated (FC > 2 and adjusted *P* < 0.05) and 154 downregulated (FC < 0.5 and adjusted *P* < 0.05) DEGs using WebGestalt (RRID:SCR_006786; ref. 21) to detect significantly altered HALLMARK gene sets (*P* < 0.05). The pathway bars were plotted using SRplot (http://www.bioinformatics.com.cn/srplot; RRID: SCR_025904).

### Protein expression datasets and analysis

Of the 37 cell lines analyzed, protein array data for 23 synergistic and nine nonsynergistic cell lines were available from DepMap (https://depmap.org/portal/data_page/?tab=customDownloads; RRID: SCR_017655). To identify differentially expressed proteins, DepMap Data Explorer 2.0 (https://depmap.org/portal/data_explorer_2/; RRID: SCR_017655) was used to plot the reverse-phase protein array (RPPA) protein levels in these cell lines. Mass spectrometry (MS) data (22), which included the protein expression data for 19 synergistic and nine nonsynergistic cell lines, were downloaded from DepMap (https://depmap.org/portal/data_page/?tab=customDownloads; RRID: SCR_017655). Protein expression levels in synergistic and nonsynergistic lines were compared and an unpaired *t* test was performed using GraphPad Prism 10 (RRID: SCR_002798).

### Co-expression network analysis

To determine the co-expressed gene modules corresponding to different PARP7i and AHRa response, the most variable 1000 genes between all 27 synergistic and 10 nonsynergistic cell lines were subjected to weighted gene co-expression network analysis (WGCNA; ref. 23) using iDEP2.10 (RRID: SCR_027373; ref. 14). Transcription factors (TF) that drove the top differential module 3 identified from WGCNA were predicted using the TRANSFAC gene set source in g:Profiler (RRID: SCR_006809; ref. 24).

### Genetic alterations in cell lines

Damaging mutation data were obtained from DepMap (https://depmap.org/portal/data_page/?tab=customDownloads; RRID: SCR_017655), and DepMap Data Explorer 2.0 (https://depmap.org/portal/data_explorer_2/; RRID: SCR_017655) was employed to plot the mean mutation allele frequencies of 18,191 genes across 27 synergistic lines and 10 nonsynergistic lines. Genes enriched for damaging mutations in the synergistic versus nonsynergistic lines were identified by comparing the mean mutation frequencies. Based on this analysis, *TP53* was selected for further investigation because it showed the highest enrichment of damaging mutations in synergistic lines versus nonsynergistic cell lines.

The mutational status of *TP53* and the levels of *TP53* mRNA [log_2_ (transcripts per million + 1); Expression Public 25Q2] were downloaded from DepMap (https://depmap.org/portal/data_page/?tab=customDownloads; RRID: SCR_017655). Sensitivity data for idasanutlin were obtained from PRISM Repurposing Public 24Q2 (the lower the value, the more sensitive) in DepMap Data Explorer 2.0 (https://depmap.org/portal/data_explorer_2/; RRID: SCR_017655). The HALLMARK_p53 pathway score was calculated by ssGSEA using GenePattern (RRID: SCR_003201; ref. 19). All data were visualized in a heatmap using Morpheus (https://software.broadinstitute.org/morpheus; RRID: SCR_014975).

### Correlation of the biomarker and immune cell infiltration in human cohorts

To evaluate the relationship between the expression of biomarker genes and immune cell infiltration, we utilized the Gene Set Cancer Analysis (GSCA) platform (https://guolab.wchscu.cn/GSCA/#/; ref. 25). Spearman correlation analysis was performed to measure the association between the mRNA levels of each biomarker gene and the abundance of various immune cell types in The Cancer Genome Atlas (TCGA)-breast invasive carcinoma (BRCA) and TCGA-prostate adenocarcinoma (PRAD) datasets (RRID: SCR_003193). Spearman correlation coefficients ranging from 0.4 to 0.7 or −0.4 to −0.7 were considered indicative of moderate correlation, whereas coefficients greater than 0.7 or below −0.7 were classified as strong correlations.

### Single-cell RNA-seq analysis

To investigate the cell-type–specific expression of the two biomarker genes, publicly available single-cell RNA-seq (scRNA-seq) data from the Broad Institute Single Cell Portal (RRID: SCR_014816) were analyzed, including data from breast cancer (https://singlecell.broadinstitute.org/single_cell/study/SCP1039; ref. 26) and prostate cancer (https://singlecell.broadinstitute.org/single_cell/study/SCP1415/cryopreservation-of-human-cancers-conserves-tumour-heterogeneity-for-single-cell-multi-omics-analysis?label=B-cells&cluster=PC-P1%20%28PID17267%29&spatialGroups=--&annotation=CellType--group--study&subsample=all; ref. 27) cohorts. The dot plot showing the expression levels of biomarker genes across annotated cell types was generated using the Broad Institute Single Cell Portal (RRID: SCR_014816).

### Survival analysis

Overall survival (OS) of patients with cancer receiving anti–PD-L1 or anti–cytotoxic T-lymphocyte–associated protein 4 (CTLA-4) treatment was analyzed using the clinical datasets (28) in the Kaplan–Meier plotter (RRID: SCR_018753). For each patient, the mean expression value of the two biomarker genes was calculated to generate a composite biomarker score. Patients were then stratified into high- and low-expression groups based on the median biomarker score. The differences in survival between high- and low-score patients with cancer receiving ICI agents were quantified by hazard ratio (HR) with log-rank *P* values derived from the Kaplan–Meier plotter (RRID: SCR_018753).

To determine whether the prognostic performance of the biomarker score was independent of clinical variables, we also used the Kaplan–Meier plotter (RRID: SCR_018753) to calculate HRs across different clinical cohorts, stratified by gender and cancer type. The forest plot was generated using SRplot (http://www.bioinformatics.com.cn/srplot; RRID: SCR_025904).

To assess the performance of immune infiltration biomarkers to predict survival in patients with cancer, marker genes of the top five infiltrated immune cells from Fig. 4A were obtained from ImmuCellAI (29) and the mean expression value of the signature genes was calculated for each immune cell type in individual patients to derive a score. Patients were subsequently categorized into high- and low-expression cohorts according to the median score. Differences in OS between the high- and low-score groups treated with anti–PD-L1 or anti–CTLA-4 were evaluated in clinical datasets (28) available in the Kaplan–Meier plotter (RRID: SCR_018753). HRs and log-rank *P* values were also calculated by the Kaplan–Meier plotter (RRID: SCR_018753).

### Data analysis

**Figure 1.**
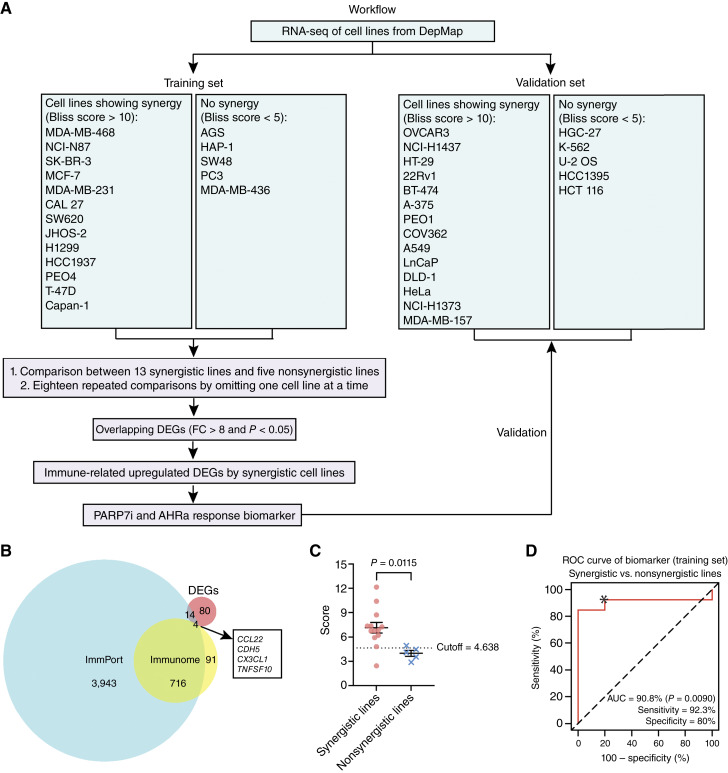
Identification of an immune-related PARP7i and AHRa response biomarker in cancer cell lines. **A,** Overview of the workflow to develop an immune-related PARP7i and AHRa response biomarker. RNA-seq data from DepMap were used to identify DEGs between cell lines showing synergistic growth inhibition by PARP7i and AHRa combination treatment and those showing no synergy. The subset of immune-related DEGs (FC > 8; *P* < 0.05) was used to define an mRNA-based biomarker to predict PARP7i and AHRa combination response. **B,** Venn diagram showing the intersection of DEGs in the training set and two lists of immune genes, ImmPort and Immunome. **C,** Scatter plot showing biomarker scores of synergistic and nonsynergistic cell lines in the training set. Biomarker scores were calculated as the average mRNA expression level of *CCL22* and *TNFSF10*. A dashed line indicates the cutoff used to classify synergistic and nonsynergistic groups. A Student *t* test was used to calculate statistical significance. **D,** ROC curve showing the performance of the two-gene classifier in distinguishing synergistic responses to PARP7i and AHRa in the training set of cell lines. The dashed diagonal line marks the performance of a random signature. The asterisk marks sensitivity and specificity selected to calculate the cutoff score to classify synergy vs. non-synergy. AUC, sensitivity, and specificity are indicated.

**Figure 2.**
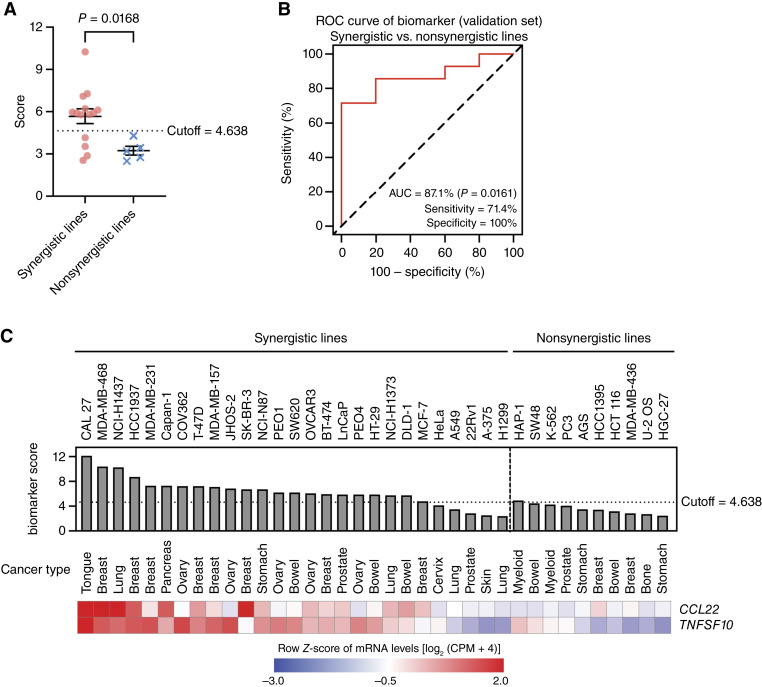
The two-gene biomarker predicts PARP7i and AHRa response in additional cancer cell lines. **A,** Scatter plot showing biomarker scores of synergistic and nonsynergistic cell lines in the validation set. Biomarker scores were calculated as the average mRNA expression of *CCL22* and *TNFSF10*. A dashed line indicates the cutoff used to distinguish between synergistic and nonsynergistic groups. A Student *t* test was used to calculate statistical significance. **B,** ROC curve showing the performance of the two-gene classifier in distinguishing synergistic responses to PARP7i and AHRa in the validation set of cell lines. The dashed diagonal line represents the performance of a random classifier using the 4.638 cutoff determined in the training set (Fig. 1C and D). AUC, sensitivity, and specificity are indicated. **C,** Bar plots of the biomarker scores in each of the 27 synergistic cell lines and the 10 nonsynergistic cell lines in the training and validation sets. A horizontal dotted line marks the optimized cutoff of 4.638. Bottom, heatmaps display the relative mRNA expression of each of the two genes comprising the transcriptional biomarker. The scale bar shows the row *Z*-score of mRNA levels for each gene. Tissue of origin for each cell line is also indicated.

Unless indicated otherwise, all data are presented as mean ± SEM. Two-sided *P* values less than 0.05 were considered statistically significant. The workflow to develop the biomarker in Fig. 1A was made in Adobe Illustrator (RRID: SCR_010279). Dot plots in Figs. 1C and 2A and Supplementary Fig. S3B were generated and an unpaired *t* test was performed using GraphPad Prism 10 (RRID: SCR_002798). Heatmaps were generated using Morpheus (https://software.broadinstitute.org/morpheus; RRID: SCR_014975). Further formatting was done in Adobe Illustrator (RRID: SCR_010279). All datasets and bioinformatic tools used are cited in the Materials and Methods section.

## Results

### A gene expression–based biomarker predicts PARP7i and AHRa combination response in cancer cells

We previously demonstrated a synergistic inhibitory effect of PARP7i and AHRa across a wide range of cancer cell lines. In a subset of hormone receptor–positive prostate and breast cancers, this therapeutic combination degraded AR and ER, respectively, and led to cell death. However, how this combination broadly suppresses cancer cell growth is unknown. Notably, Bliss synergy scores for the PARP7i and AHRa combination were not correlated with mRNA levels of *PARP7* (Supplementary Fig. S1A) or *AHR* (Supplementary Fig. S1B). They were also not correlated with the ssGSEA-based AHR pathway score (Supplementary Fig. S1C). These findings highlight the importance of understanding the determinants of response and defining a predictive response biomarker to accelerate the utility of this combination therapy.

To begin to define the molecular characteristics of response, we analyzed RNA-seq data of synergistic and nonsynergistic cell lines. In a prior study, we measured the synergistic effect of PARP7i and AHRa across a panel of 47 cancer cell lines (9). Of these, 38 cell lines had available RNA-seq data in the DepMap database. From this group, 37 cell lines were selected for subsequent analysis, including 27 synergistic lines (Bliss score >10) and 10 nonsynergistic lines (Bliss score <5). This subset was randomly divided into training and validation sets (Fig. 1A). Differential expression analysis of the 13 synergistic and five nonsynergistic cell lines in the training set identified 236 genes that were upregulated (FC > 8 and *P* < 0.05) in the synergistic lines compared with the nonsynergistic lines (Supplementary Table S1). To ensure the robustness of our signature, an LOO analysis was performed by iteratively omitting one cell line at a time and repeating the DEG analysis. This approach yielded 98 consistently upregulated genes shared across all comparisons (Supplementary Table S1).

Because PARP7 and AHR are involved in immune regulation (1, 5, 10, 11) and PARP7i has been evaluated in combination with ICI therapies (NCT05127590; ref. 1), we aimed to develop a PARP7i and AHRa combination signature with relevance to immune response. To this end, we intersected the list of 98 significantly upregulated genes with two immune-related gene sets from ImmPort and Immunome, identifying four overlapping immune-related genes: *CCL22*, *CDH5*, *CX3CL1*, and *TNFSF10* (Fig. 1B). ROC analysis of each gene revealed that *CCL22* and *TNFSF10* significantly predicted synergy, with AUC values exceeding 80% (*P* < 0.05; Supplementary Fig. S1D). In contrast, *CDH5* and *CX3CL1* had AUCs below 80% and did not reach statistical significance (*P* > 0.05; Supplementary Fig. S1E). *CDH5* and *CX3CL1* were, therefore, excluded from further consideration.

Averaging the expression values of genes is a commonly used method to quantify a composite gene expression signature associated with a particular biological activity (30–32). In our analysis, differential expression profiling identified two genes, *CCL22* and *TNFSF10*, which were most strongly associated with the synergistic response to PARP7i and AHRa. Given the limited number of genes, we defined a two-gene expression biomarker based on the mean expression of *CCL22* and *TNFSF10* rather than a broader gene signature. Synergistic cell lines showed significantly higher biomarker scores compared with nonsynergistic lines in our training set (Fig. 1C). The biomarker also significantly distinguished synergistic cell lines from nonsynergistic cell lines, with an AUC of 90.8% at 92.3% sensitivity and 80% specificity (Fig. 1D), indicating a 90.8% probability that the score of a randomly chosen synergistic cell line is higher than that of a randomly chosen nonsynergistic line. The optimal cutoff point was defined using LR to maximize both sensitivity and specificity (*cutoff = 4.638; ref. 18).

To validate this two-gene biomarker, we tested its ability to predict the response of the 14 synergistic and five nonsynergistic cell lines in the validation set (Fig. 1A). In this independent cohort of cells, the biomarker scores of synergistic cell lines were significantly increased compared with those of nonsynergistic lines (Fig. 2A). Based on the cutoff determined by the training set, the validation set showed a sensitivity of 71.4% and a specificity of 100% for an AUC of 87.1% (Fig. 2B), indicating that the biomarker can robustly predict response. Despite some variations in the expression level of each individual genes, 22 of 27 synergistic cell lines in the training and validation sets had scores above the cutoff (Fig. 2C). The scores of nine of 10 nonsynergistic cell lines fell below the cutoff, indicating good specificity (Fig. 2C).

To test the necessity of deriving a multigene biomarker, we performed ROC analysis for each individual gene using data from all 37 cell lines. Neither the AUC of *CCL22* (80%) nor that of *TNFSF10* (84.4%) exceeded the AUC of the combined two-gene biomarker (86.7%; Supplementary Fig. S2A–S2C), highlighting the value of using a multigene biomarker rather than a single gene to predict response. To further confirm the functional independence, we examined the expression of *CCL22* and *TNFSF10* across 1,684 cell lines in DepMap cell lines and found no significant correlation in their expression, suggesting that these genes are not co-regulated (Supplementary Fig. S2D). Because a multigene biomarker is more robust than a single gene in complex biological systems, we opted to use the expression of both transcripts to define a genetic biomarker for PARP7i and AHRa response.

In addition to a binary classification of synergy, we also performed correlation analysis of Bliss score and biomarker score to evaluate synergy as a continuous variable. These two scores showed a moderate but significant correlation (Pearson r = 0.54 and *P* = 0.0006; Spearman r = 0.48 and *P* = 0.0027; Supplementary Fig. S2E), further supporting the validity of the biomarker in predicting the response to PARP7i and AHRa combination therapy.

### Molecular characteristics of synergistic response

**Figure 3.**
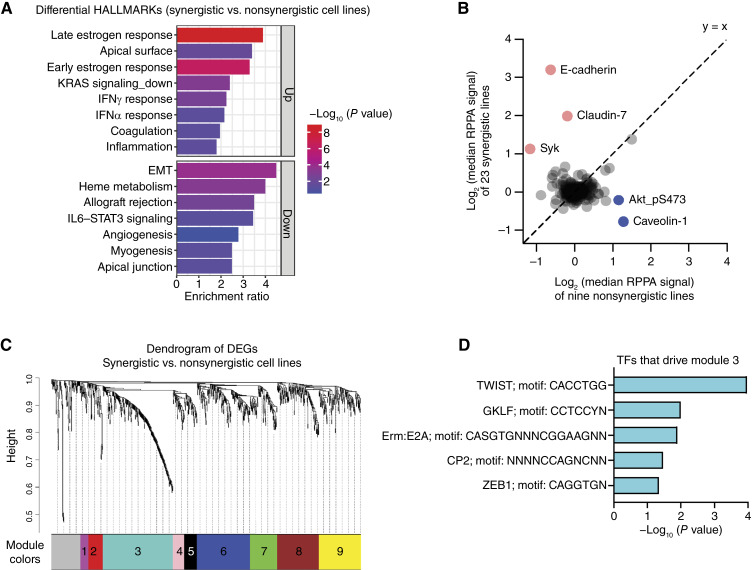
Synergistic cancer cells have unique transcriptional and proteomic characteristics. **A,** Bar plot of HALLMARK pathways (*P* < 0.05) identified in ORA of 588 upregulated (FC > 2 and adjusted *P* < 0.05) and 154 downregulated (FC < 0.5 and adjusted *P* < 0.05) genes in synergistic cell lines compared with nonsynergistic cell lines. **B,** Scatter plot showing the median values of RPPA protein expression levels in synergistic vs. nonsynergistic cell lines. Proteins with higher expression in synergistic cell lines are highlighted in red whereas those more highly expressed in nonsynergistic lines are shown in blue. **C,** Dendrogram of co-expressed gene modules identified by WGCNA using the 1,000 most variable genes across 27 synergistic and 10 nonsynergistic cell lines. Genes were grouped into nine distinct modules based on expression similarity. Unassigned genes are shown in gray. **D,** Bar plots showing the five TFs most significantly associated with the regulation of module 3. TFs are ranked by *P* value with the most significant shown on the top.

To define transcriptional characteristics specific to synergistic cell lines that may provide mechanistic insights, we compared the transcriptional profiles of the 27 synergistic and 10 nonsynergistic cell lines in both the training and validation sets. These comparisons identified 588 upregulated (FC > 2 and adjusted *P* < 0.05) and 154 downregulated (FC < 0.5 and adjusted *P* < 0.05) DEGs. These lists of DEG were then subjected to ORA to assess whether genes associated with specific biological process were enriched or overrepresented in the synergistic cell lines (33). This analysis revealed that cell lines showing synergy were enriched for immune-related HALLMARK pathways, including IFNα/γ response and inflammation compared with nonsynergistic lines (Fig. 3A). Synergistic cell lines also showed enrichment for hormone receptor–related gene sets, including early/late ER (Fig. 3A), likely because of the high number of ER-positive cell lines among the synergistic cell lines. Synergistic cell lines also showed downregulation of EMT pathway genes (Fig. 3A).

To explore whether these transcriptional characteristics were reflected at the protein level, we next analyzed the protein array data available for these cell lines in DepMap. Consistent with the gene expression data, cells that responded to AHRa and PARP7i also expressed higher levels of epithelial markers, including E-cadherin, Claudin-7, and Syk and lower levels of Akt_pS473 and Caveolin-1 proteins (Fig. 3B; Supplementary Fig. S3A). Notably, Akt_pS473 and Caveolin-1 are known activators of the PI3K/Akt signaling pathway, which promotes proliferation and EMT (34).

To cross-validate the protein array findings, we analyzed the corresponding proteins in the MS dataset from DepMap (22). E-cadherin (*CDH1*) was consistently enriched in synergistic cell lines across both the protein array and MS datasets. Claudin-7, Syk, and Caveolin-1, however, were only differentially expressed in the protein array data and Akt-pS473 was not detected in the MS data (Supplementary Fig. S3B).

We next compared the predictive performance of the identified pathways and protein markers characteristic of synergistic cell lines against our gene expression biomarker. The HALLMARK_IFNα/γ response and HALLMARK_EMT pathways had limited predictive ability to distinguish synergistic and nonsynergistic cell lines with AUC values around 60% and *P* > 0.05 (Supplementary Fig. S3C). Similarly, none of the epithelial proteins (E-cadherin, Claudin-7, Syk, Caveolin-1, and Akt_pS473) or their corresponding mRNAs (*CDH1*, *CLDN7*, *SYK*, and *CAV1*) outperformed the two-gene biomarker in ROC analysis (Supplementary Fig. S3D and S3E), providing further support that our two-gene biomarker performs better than signatures of the IFN or EMT pathway in predicting response.

To gain a deeper understanding of transcriptional features underlying synergy response, we next performed co-expression network analysis (35) to identify gene modules associated with response to PARP7i and AHRa combination therapy. This approach clusters genes into functionally related modules and provides a system-level view that can reveal key regulators and biologically relevant pathways that can be missed by single-gene analyses (35). Using the 1,000 most variable transcripts between synergistic and nonsynergistic lines, we identified nine distinct modules composed of highly co-expressed genes (Fig. 3C). Approximately one third of assigned genes mapped to module 3 (Fig. 3C), prompting us to investigate the molecular mechanisms controlling the expression of genes in this module. Functional enrichment using g:Profiler (24) identified binding sites of several TFs, including TWIST, GKLF, E2A, CP2, and ZEB1 (Fig. 3D), raising the possibility that these TFs may drive the different gene expression patterns observed between synergistic and nonsynergistic lines. Notably, TWIST is a known inducer of EMT (36), aligning with the observed differential activation of the EMT pathway between the groups (Fig. 3A).

We next asked whether cancer cell lines showing different response to PARP7i and AHRa carried specific genetic alterations. Using mutation data available in DepMap, we analyzed the mutational status of 18,191 genes across all 37 cell lines in our training and validation sets. This analysis revealed a slightly higher frequency of *TP53* mutations in synergistic lines compared with nonsynergistic lines (Supplementary Fig. S4A). However, *TP53* mRNA expression levels, the activity of HALLMARK_p53 pathway, which includes genes involved in p53 signaling and serves as a comprehensive indicator of p53 activity, and sensitivity to idasanutlin, an MDM2 inhibitor that stabilizes p53 by blocking its degradation (37), were not associated with synergy (Supplementary Fig. S4B).

### PARP7i and AHRa response biomarker is associated with benefit from immune checkpoint inhibitors

**Figure 4.**
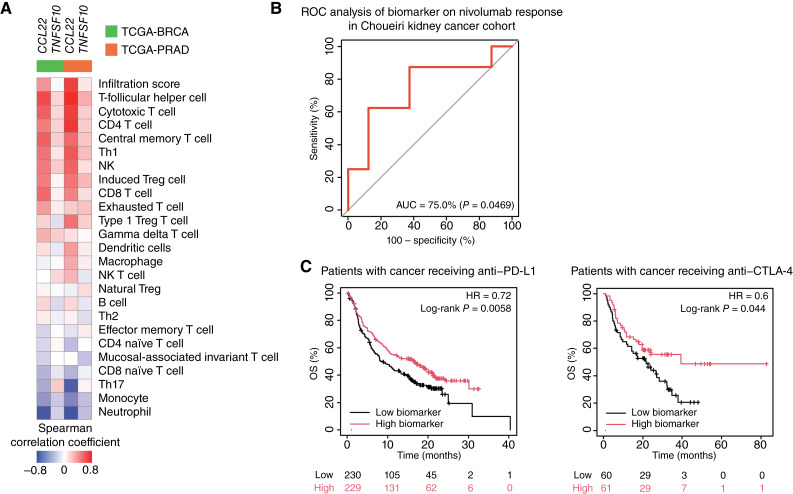
PARP7i and AHRa response biomarker is associated with response to ICI therapy. **A,** Heatmap showing Spearman correlation coefficients between the two biomarker genes and immune cell types in TCGA-BRCA and TCGA-PRAD patient data. **B,** ROC curve showing the performance of the two-gene classifier in distinguishing response to the anti–PD-1 antibody, nivolumab, in kidney cancer. The AUC represents the probability that a randomly selected anti–PD-1–responsive patient will have a higher biomarker score than a randomly selected nonresponsive patient. **C,** Kaplan–Meier survival curves showing the OS of patients with cancer receiving anti–PD-L1 (left) or anti–CTLA-4 treatment (right). Patients were stratified into “high” and “low” groups based on the median value of the PARP7i and AHRa response biomarker scores. Patients with biomarker scores above than median were classified as “high” and those below as “low.” HR and *P* values are included on each plot. The numbers of surviving patients at each timepoint in the “high” and “low” groups are indicated below each plot.

Because our gene expression biomarker was associated with immune pathway activation in cell line models (Fig. 3A), we next investigated the immune characteristics of the biomarker in patient tumors. The correlation of each biomarker gene and immune cell infiltration was determined for TCGA-BRCA and TCGA-PRAD samples. *CCL22* demonstrated moderate-to-strong positive correlations with immune cells, including NK, T-follicular helper cell, CD4^+^ T cell, cytotoxic T cell, central memory T cell, Th1, induced regulatory T cell (Treg), and CD8 T cells. In contrast, *CCL22* expression was negatively correlated with neutrophil and Th17 cells. *TNFSF10* had a weaker correlation with immune cell infiltration than *CCL22* (Fig. 4A). To determine whether the association between the two genes comprising our response biomarker and immune infiltration in TCGA bulk tissues was driven by higher expression of these genes in immune cells rather than cancer cells, we examined the expression patterns of *CCL22* and *TNFSF10* using publicly available scRNA-seq datasets of breast (26) and prostate (27) cancers from the Broad Institute Single Cell portal. Our analysis revealed that *CCL22* was expressed at low levels across all cell types, whereas *TNFSF10* exhibited higher expression in cancer cells compared with other cell types in the tumor microenvironment (Supplementary Fig. S5A). These findings suggest that the elevated biomarker scores observed in bulk tissue are likely attributable to tumor-intrinsic transcriptional activity rather than expression from infiltrating immune cells.

With the positive correlation between our PARP7i/AHRa response biomarker and immune infiltration, we next asked whether the expression of the biomarker genes was correlated with clinical response to immunotherapy treatments. Using transcriptomic data available from ClinicalOmicsDB (20), we measured the association between *CCL22* and *TNFSF10* mRNA expression and patient response to single-agent ICI therapy. In a trial that tested an anti–PD-1 antibody, nivolumab, in patients with kidney cancer (Choueiri cohort; ref. 38), the *CCL22*- and *TNFSF10*-based score differentiated the response at an AUC of 75% (Fig. 4B). We also examined the prognostic value of this biomarker in patients with cancer who received ICI therapies. Patients with high (> median) scores who received anti–PD-L1 and anti–CTLA-4 therapies had significantly better OS than those with a low score (< median; Fig. 4C).

We then asked how each individual gene performed compared with the composite score in predicting the response to nivolumab. *CCL22* alone achieved an AUC of 79.7% (*P* = 0.0102), which was slightly higher than that of the two-gene biomarker (75%; *P* = 0.0469). *TNFSF10* had a lower AUC of 60.9% (*P* = 0.4492), suggesting weaker predictive power on its own (Supplementary Fig. S5B). We further evaluated the prognostic value of these genes in independent cohorts of patients receiving ICI therapies. In patients receiving anti–PD-L1 (Supplementary Fig. S5C), *CCL22* and *TNFSF10* were associated with HRs of 0.73 and 0.78, respectively, which were comparable with the two-gene biomarker score (HR = 0.72). In patients treated with anti–CTLA-4 (Supplementary Fig. S5D), each individual gene again showed similar performance to the two-gene biomarker score (*CCL22*, HR = 0.62; *TNFSF10*, HR = 0.59; and two-gene score, HR = 0.60). Despite weaker performance in the Choueiri cohort (Supplementary Fig. S5B), *TNFSF10* showed good prognostic value in multiple ICI-treated cohorts (Supplementary Fig. S5C and S5D). Therefore, we retained both genes to provide greater predictive power, robustness, and generalizability.

To evaluate whether the prognostic value of the *CCL22* and *TNFSF10* score was independent of known clinical variables, we assessed the biomarker in different clinical cohorts, stratified by gender and cancer type. In patients treated with an anti–CTLA-4 therapy, the biomarker score remained predictive of OS across clinical subgroups, including male, female, and melanoma (HRs <1; Supplementary Fig. S5E), suggesting that its prognostic value is likely robust in these cohorts. In the cohort of patients treated with anti–PD-L1 therapy, the biomarker demonstrated partial independence, however, in certain clinical subgroups, such as male and urothelial cancer, and HRs were more than 1 (Supplementary Fig. S5E). Analysis in other cancer types was not feasible because of low sample numbers.

To investigate whether the performance of our biomarker could be attributable solely to its association with immune infiltration, we analyzed the signatures of the top five infiltrated immune cells from Fig. 4A to predict OS of patients with cancer receiving ICIs. In patients receiving anti–PD-L1, T-follicular helper cell, cytotoxic T cell, and CD4 T cell predicted OS with HRs of 0.66, 0.62, and 0.66, respectively, which were lower than the HR of our two-gene biomarker score (HR: 0.72). Central memory T cell and Th1 exhibited higher HRs of 0.83 and 0.74, respectively (Supplementary Fig. S6A). In patients receiving anti–CTLA-4, T-follicular helper cell, cytotoxic T cell, central memory T cell, and Th1 predict the OS at lower HRs (0.66, 0.35, 0.6, and 0.56, respectively) than the biomarker, whereas CD4 T cells had a slightly higher HR (0.73; Supplementary Fig. S6A).

We also evaluated the predictive performance of several immune markers for ICI response using the Choueiri Kidney Cancer Cohort in the ClinicalOmicsDB database (20). The AUCs for CIBERSORT.ABS_T_cell_follicular_helper, EPIC_T_cell_CD4^+^, MCP.COUNTER_T_cell, and ESTIMATE_immune_score were 49.3%, 31.3%, 76.6%, and 79.7%, respectively (Supplementary Fig. S6B).

## Discussion

We previously demonstrated that combining PARP7i and AHRa can extend the growth-inhibitory effects of these drugs to a broader range of tumor lines (9). Although many cell lines exhibited limited sensitivity to PARP7i or AHRa monotherapy, a subset showed a marked sensitivity to the combination (9). As response was not correlated with the tissue of origin or levels of *PARP7* or *AHR*, we undertook a multiomics approach to map determinants of response and to define a gene expression biomarker predictive of response to the combination of PARP7i and AHRa. The results of these analyses identified a two-gene expression score that captures a common transcriptional program predictive of PARP7i and AHRa synergistic lethality across tumor types. In the absence of clinical trial data evaluating the combination therapy that would allow direct validation of the biomarker’s predictive ability, we instead leveraged multiple publicly available datasets of human patients with cancer to further characterize the biological and clinical features associated with this two-gene expression score.

The transcriptional biomarker score we developed is based on the expression of two immune-related genes, *CCL22* and *TNFSF10*, each playing distinct roles in immune regulation and tumor biology. *CCL22* encodes C–C motif chemokine 22, a ligand for the chemokine receptor CCR4. It is known to be a chemotactic for several immune cell types, including monocytes, dendritic and NK, and activated T cells (39). In the tumor microenvironment, CCL22 has been implicated in recruiting Tregs, which can suppress antitumor immune response and contribute to immune evasion (39, 40). *TNFSF10*, also known as TRAIL (TNF-related apoptosis-inducing ligand), encodes a member of the TNF superfamily that can selectively induce apoptosis in cancer cells upon binding to its receptors (41, 42). Although both genes are involved in immune-related processes, they operate in distinct biological pathways and are expressed by different cell types.

The integration of transcriptomic and proteomic data further defined the molecular basis of PARP7i and AHRa response. RPPA data revealed an enrichment of epithelial proteins in synergistic cell lines, suggesting opportunities to further refine the biomarker by incorporating a protein biomarker or protein biomarker combinations. Although we found discrepancies between protein array and MS datasets, E-cadherin, the top enriched protein in the RPPA data, was consistently elevated in synergistic lines in the MS dataset as well. These differences are likely attributable to inherent technical differences between the two platforms—MS offers broader but sometimes lower sensitivity for specific proteins, whereas antibody-based arrays may be more sensitive to certain targets but are limited by antibody specificity and availability. These observations underscore the benefits of performing multiomics analysis to obtain a comprehensive understanding of the biological mechanisms that mediate drug response (43).

Pathway enrichment analysis of significant DEGs between all synergistic and nonsynergistic cell lines revealed that multiple immune-related gene sets were upregulated in cells showing synergy, including IFNα/γ response and inflammation (Fig. 3A). This observation suggests that immune activation may sensitize cells to the combination of PARP7i and AHRa. In contrast, EMT, heme metabolism, and allograft rejection pathways were downregulated in synergistic cell lines (Fig. 3A), suggesting that suppression of these pathways might enhance responsiveness to the combination therapy. Finally, we observed an enrichment of hormone receptor–related pathways, including ER (Fig. 3A), which may be attributable to the inclusion of ER-positive cell lines in the analysis.

To obtain further insights into the biological nature of different PARP7i and AHRa responses, we explored genetic alterations across both synergistic and nonsynergistic cell lines. Mutations in *TP53*, which encodes the tumor suppressor p53, were slightly more common in synergistic than in nonsynergistic cell lines. In concordance with this observation, AHR and p53 may regulate common downstream targets, and the antitumorigenesis effect of AHRa may depend upon p53 (44–46). Moreover, alterations in p53 are known to reshape the immune microenvironment, leading to changes in chemokine/cytokine secretion, myeloid cells, and T cells (47, 48). Therefore, *TP53* mutations may also contribute to the elevated expression of immune-related genes observed in synergistic lines. However, further investigation is needed to establish a causal relationship.

Although the individual roles of PARP7 and AHR in modulating tumor vulnerability to ICI have been studied (1, 12), the impact of combining PARP7i and AHRa on immune response has not been explored. In this study, we compared immune-related DEGs between cells that showed a synergistic response to PARP7i and AHRa with those that did not to develop a transcriptional biomarker for synergistic response. Notably, we found that this biomarker was also associated with response to single agent ICI. Overall, ICI therapies have shown limited efficacy in many solid tumors, largely because of the high prevalence of immunogenically “cold” tumors that lack sufficient numbers of tumor-infiltrating lymphocytes. In TCGA-BRCA and TCGA-PRAD patient datasets, both *CCL22* and *TNFSF10 *expression were positively correlated with tumor infiltration of some immune cell types (Fig. 4A). The biomarker score was also associated with ICI therapy response (Fig. 4B) and OS, with better OS observed in high-scoring patients and worse OS in low-scoring patients who received various types of ICI agents (Fig. 4C).

It is important to note that our biomarker score was specifically developed to identify patients who might benefit from the combination of PARP7i and AHRa, rather than to broadly predict ICI response across cancer types or immune-infiltrated tumors. Therefore, we do not expect it to universally outperform established immune infiltration. Nonetheless, our data demonstrate that the *CCL22*- and *TNFSF10-*based score offers predictive benefits over some, although not all, markers of immune infiltration (Supplementary Fig. S6A and S6B). The ability of this biomarker to predict ICI response might be attributed to the intricate interplay between AHR signaling and immune regulation as AHR shapes both innate and adaptive immune responses (10, 11).

A key limitation of our study is the lack of clinical data from patients treated with a PARP7i and AHRa combination. Consequently, the validation of the biomarker was limited to an independent cell line dataset rather than patient samples. In addition, because of limited number of cell lines tested, the training and validation sets had an imbalance of cancer types. To alleviate potential bias from individual cell lines, we conducted an LOO analysis to establish the robustness of the biomarker. Because some cancer types may be inherently more sensitive to the combination, further analysis across additional cell line models with a more balanced sampling of cancer types is needed to robustly assess potential tissue-of-origin effects. We also observed that some variables, such as sex and cancer type, partially influenced the prognostic performance of the biomarker (Supplementary Fig. S5E). These results may reflect true biological differences or may be affected by limited sample size.

In conclusion, our multiomic characterization has identified potential biomarkers to stratify tumors for benefit from the combination of PARP7i and AHRa agents and explored possible underlying molecular mechanisms. Further validation of the predictive power of this transcriptional biomarker across diverse preclinical models and retrospective patient cohorts will be needed to establish its clinical relevance. Additionally, mechanistic studies to elucidate how the PARP7i and AHRa combination modulates the tumor immune microenvironment will be crucial for optimizing therapeutic strategies and identifying additional biomarkers of response.

## Supplementary Material

Supplementary Table S1 Supplementary Table S1. The list of upregulated DEGs from the comparison between 13 synergistic lines and 5 non-synergistic lines and 18 LOO comparisons.

Supplementary Figure S1 Supplementary Figure S1, related to Figure 1. Identification of an immune-related PARP7i and AHRa response biomarker in cancer cell lines.

Supplementary Figure S2 Supplementary Figure S2, related to Figure S2. The biomarker predicts PARP7i and AHRa response in additional cancer cell lines.

Supplementary Figure S3 Supplementary Figure S3, related to Figure 3. Synergistic cancer cells have unique transcriptional, proteomic and mutational characteristics.

Supplementary Figure S4 Supplementary Figure S4, related to Figure 3. TP53 mutation enriched in synergistic cell lines.

Supplementary Figure S5 Supplementary Figure S5, related to Figure 4. PARP7i and AHRa response signature is associated with benefit from immune checkpoint blockade.

Supplementary Figure S6 Supplementary Figure S6, related to Figure 4. PARP7i and AHRa response biomarker offers predictive benefits over some, though not all, markers of immune infiltration.

## Data Availability

The data analyzed in this study were obtained from DepMap at https://depmap.org/portal/data_page/?tab=allData (RRID: SCR_017655), InnateDB at https://www.innatedb.com/ (RRID: SCR_006714), ClinicalOmicsDB at https://trials.linkedomics.org/, GSCA platform at https://guolab.wchscu.cn/GSCA/#/, Broad Institute Single Cell Portal (RRID: SCR_014816) at https://singlecell.broadinstitute.org/single_cell/study/SCP1039 and https://singlecell.broadinstitute.org/single_cell/study/SCP1415/cryopreservation-of-human-cancers-conserves-tumour-heterogeneity-for-single-cell-multi-omics-analysis?label=B-cells&cluster=PC-P1%20%28PID17267%29&spatialGroups=–&annotation=CellType–group–study&subsample=all, and Kaplan–Meier plotter at https://kmplot.com/analysis/index.php?p=service&cancer=immunotherapy (RRID: SCR_018753). Downstream analytic data generated in this study are available upon request from the corresponding author.
