# Supplementary material for: Identification of a Two-Gene Biomarker Correlated with Sensitivity to Combined PARP7 Inhibition and AHR Activation in Cancer Cells
Source: Cancer Res Commun. 2026 Jan 2;6(1):5–16. doi: 10.1158/2767-9764.CRC-25-0173 (PMC12757997; doi:10.1158/2767-9764.CRC-25-0173)
Supplement: Supplementary Figure S1 — , related to Figure 1. Identification of an immune-related PARP7i and AHRa response biomarker in cancer cell lines. [file crc-25-0173_supplementary_figure_s1_suppsf1.pdf]

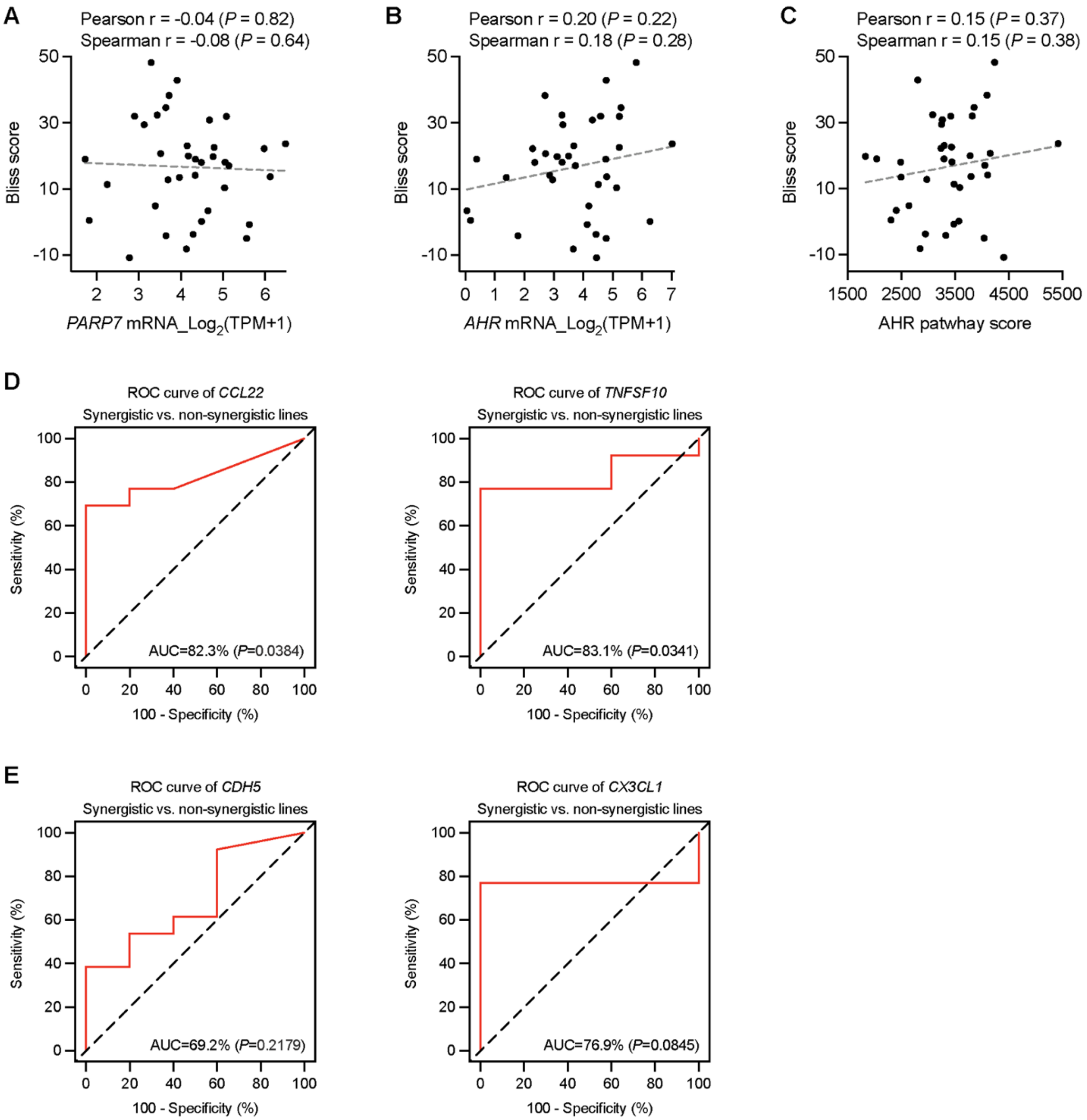

Supplementary Figure S1, related to Figure 1. Identification of an immune-related PARP7i and AHRa response biomarker in cancer cell lines.

A-C. Scatter plots showing the correlative relationship of Bliss score of 27 synergistic lines and 10 non-synergistic lines to mRNA levels of *PARP7* **(A)** and *AHR* **(B)** and AHR pathway score **(C)**.

D. ROC curves showing the performance of *CCL22* and *TNFSF10* expression in distinguishing synergistic response to PARP7i and AHRa combined treatment in the training set of cell lines.

E. ROC curves showing the performance of *CDH5* and *CX3CL1* expression in distinguishing synergistic response to PARP7i and AHRa combined treatment in the training set of cell lines.
