# Supplementary material for: Identification of a Two-Gene Biomarker Correlated with Sensitivity to Combined PARP7 Inhibition and AHR Activation in Cancer Cells
Source: Cancer Res Commun. 2026 Jan 2;6(1):5–16. doi: 10.1158/2767-9764.CRC-25-0173 (PMC12757997; doi:10.1158/2767-9764.CRC-25-0173)
Supplement: Supplementary Figure S2 — , related to Figure S2. The biomarker predicts PARP7i and AHRa response in additional cancer cell lines. [file crc-25-0173_supplementary_figure_s2_suppsf2.pdf]

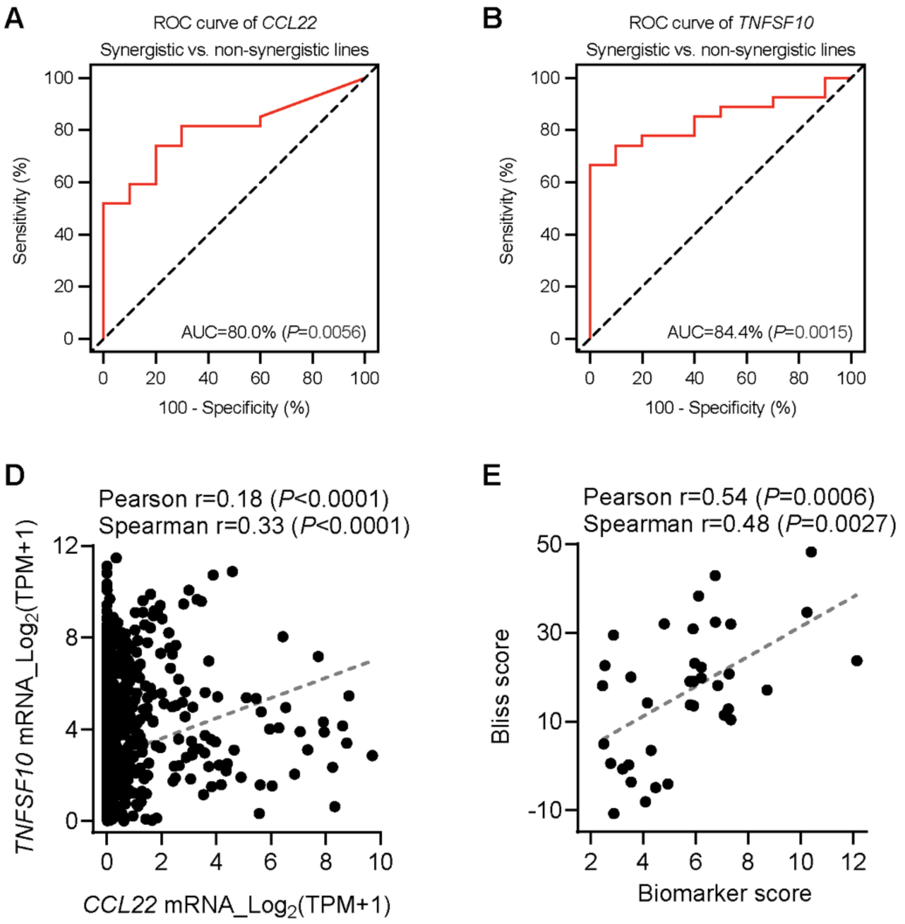

Supplementary Figure S2, related to Figure 2. The biomarker predicts PARP7i and AHRa response in additional cancer cell lines.

A-C. ROC curves showing the performance of individual biomarker genes *CCL22* (**A**) and *TNFSF10* (**B**) as well as the combined two-gene biomarker (**C**) in distinguishing synergistic vs non-synergistic cell lines across all 37 cell lines.

D. Scatter plots showing the correlative relationship of *CCL22* and *TNFSF10* mRNA expression across all 1,684 cell lines in DepMap.

E. Scatter plot showing the correlative relationship between Bliss score and biomarker score for all 37 cell lines.
