# Supplementary material for: Identification of a Two-Gene Biomarker Correlated with Sensitivity to Combined PARP7 Inhibition and AHR Activation in Cancer Cells
Source: Cancer Res Commun. 2026 Jan 2;6(1):5–16. doi: 10.1158/2767-9764.CRC-25-0173 (PMC12757997; doi:10.1158/2767-9764.CRC-25-0173)
Supplement: Supplementary Figure S3 — , related to Figure 3. Synergistic cancer cells have unique transcriptional, proteomic and mutational characteristics. [file crc-25-0173_supplementary_figure_s3_suppsf3.pdf]

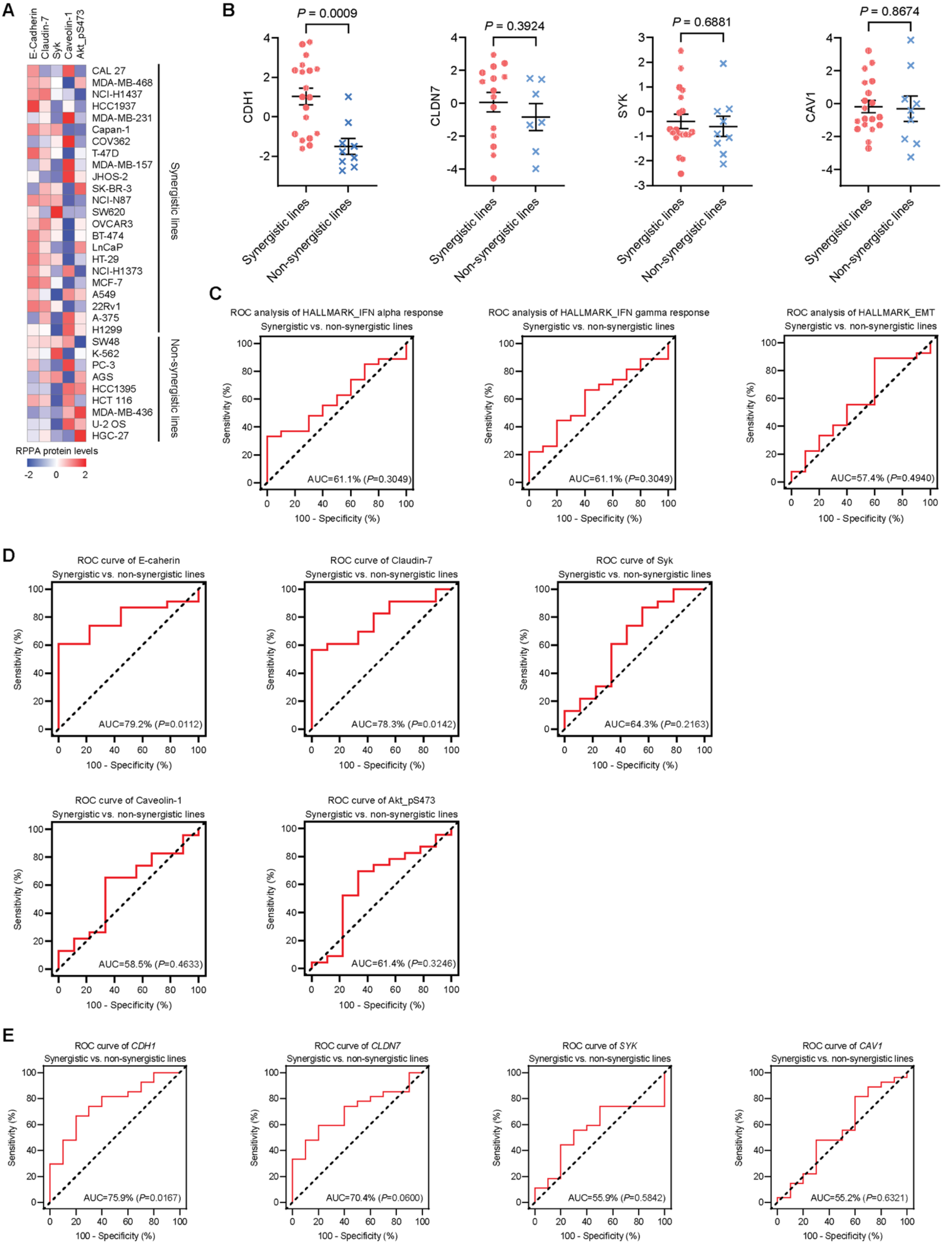

A
E-Cadherin
Claudin-7
Syk
Caveolin-1
Akt_pS473
CAL 27
MDA-MB-468
NCI-H1437
HCC1937
MDA-MB-231
Capan-1
COV362
T-47D
MDA-MB-157
JHOS-2
SK-BR-3
NCI-N87
SW620
OVCAR3
BT-474
LnCaP
HT-29
NCI-H1373
MCF-7
A549
22Rv1
A-375
H1299
SW48
K-562
PC-3
AGS
HCC1395
HCT 116
MDA-MB-436
U-2 OS
HGC-27
Synergistic lines
Non-synergistic lines
RPPA protein levels
-2
0
2
B
P = 0.0009
P = 0.3924
P = 0.6881
P = 0.8674
CDH1
CLDN7
SYK
CAV1
Synergistic lines
Non-synergistic lines
C
ROC analysis of HALLMARK_IFN alpha response
Synergistic vs. non-synergistic lines
AUC=61.1% (P=0.3049)
ROC analysis of HALLMARK_IFN gamma response
Synergistic vs. non-synergistic lines
AUC=61.1% (P=0.3049)
ROC analysis of HALLMARK_EMT
Synergistic vs. non-synergistic lines
AUC=57.4% (P=0.4940)
Sensitivity (%)
100 - Specificity (%)
D
ROC curve of E-caherin
Synergistic vs. non-synergistic lines
AUC=79.2% (P=0.0112)
ROC curve of Claudin-7
Synergistic vs. non-synergistic lines
AUC=78.3% (P=0.0142)
ROC curve of Syk
Synergistic vs. non-synergistic lines
AUC=64.3% (P=0.2163)
ROC curve of Caveolin-1
Synergistic vs. non-synergistic lines
AUC=58.5% (P=0.4633)
ROC curve of Akt_pS473
Synergistic vs. non-synergistic lines
AUC=61.4% (P=0.3246)
E
ROC curve of CDH1
Synergistic vs. non-synergistic lines
AUC=75.9% (P=0.0167)
ROC curve of CLDN7
Synergistic vs. non-synergistic lines
AUC=70.4% (P=0.0600)
ROC curve of SYK
Synergistic vs. non-synergistic lines
AUC=55.9% (P=0.5842)
ROC curve of CAV1
Synergistic vs. non-synergistic lines
AUC=55.2% (P=0.6321)

Supplementary Figure S3, related to Figure 3. Synergistic cancer cells have unique transcriptional, proteomic and mutational characteristics.

A. Heatmap showing the expression of differentially expressed proteins in 23 synergistic cell lines and 9 non-synergistic lines. The scale bar shows the row Z-score of RPPA levels of individual protein. The synergy classification for cell lines is indicated.

B. Scatter plot showing the protein levels of E-Cadherin (*CDH1*), Claudin-7 (*CLDN7*), Syk (*SYK*) and Caveolin-1 (*CAV1*) in 19 synergistic and 9 non-synergistic cell lines from the MS dataset. A Student's t-test was used to calculate statistical significance.

C. ROC curves showing the performance of the indicated HALLMARK pathways to distinguish 27 synergistic cell lines from 10 non-synergistic lines. The scores of HALLMARK_IFN alpha response, HALLMARK_IFN gamma response and HALLMARK_EMT were calculated by ssGSEA and the score represents the changes of genes in these pathways in each cell line.

D. ROC curves showing the performance of E-Cadherin, Claudin-7, Syk, Caveolin-1 and Akt_pS473 protein levels in distinguishing 23 synergistic cell lines from 9 non-synergistic lines.

E. ROC curves showing the performance of *CDH1*, *CLDN7*, *SYK* and *CAV1* mRNA levels in distinguishing 27 synergistic cell lines from 10 non-synergistic lines.
