# Supplementary material for: Identification of a Two-Gene Biomarker Correlated with Sensitivity to Combined PARP7 Inhibition and AHR Activation in Cancer Cells
Source: Cancer Res Commun. 2026 Jan 2;6(1):5–16. doi: 10.1158/2767-9764.CRC-25-0173 (PMC12757997; doi:10.1158/2767-9764.CRC-25-0173)
Supplement: Supplementary Figure S4 — , related to Figure 3. TP53 mutation enriched in synergistic cell lines. [file crc-25-0173_supplementary_figure_s4_suppsf4.pdf]

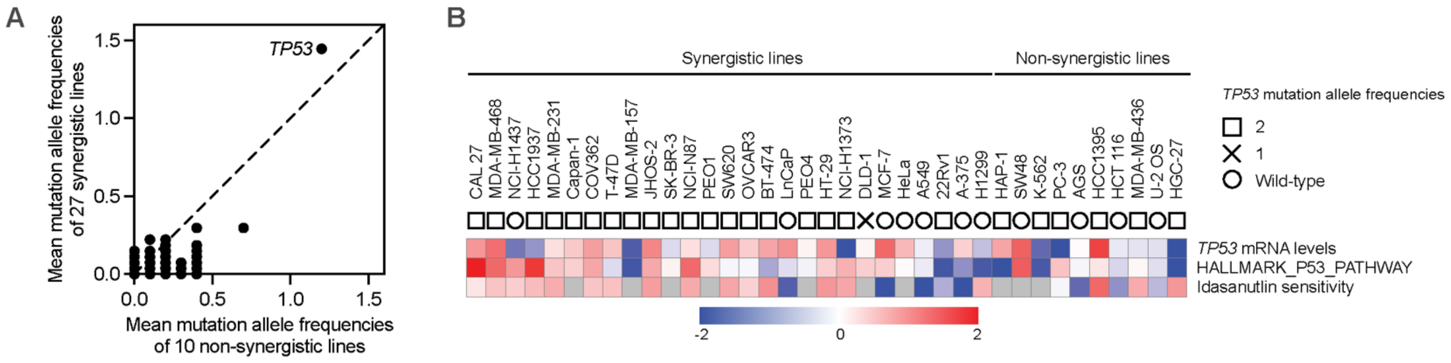

Supplementary Figure S4, related to Figure 3. *TP53* mutation enriched in synergistic cell lines.

A. Scatter plot showing frequency of damaging mutations in individual genes in 27 synergistic cell lines (y-axis) vs 10 non-synergistic lines (x-axis). The diagonal line indicates the line of equality (x = y).

B. Heatmap showing *TP53* mRNA levels, *TP53* mutation frequency, ssGSEA scores of HALLMARK_p53 pathway activity and idasanutlin sensitivity for the 27 synergistic cell lines and 10 non-synergistic lines. Scale bar indicates row Z-score.
