# Supplementary material for: Identification of a Two-Gene Biomarker Correlated with Sensitivity to Combined PARP7 Inhibition and AHR Activation in Cancer Cells
Source: Cancer Res Commun. 2026 Jan 2;6(1):5–16. doi: 10.1158/2767-9764.CRC-25-0173 (PMC12757997; doi:10.1158/2767-9764.CRC-25-0173)
Supplement: Supplementary Figure S5 — , related to Figure 4. PARP7i and AHRa response signature is associated with benefit from immune checkpoint blockade. [file crc-25-0173_supplementary_figure_s5_suppsf5.pdf]

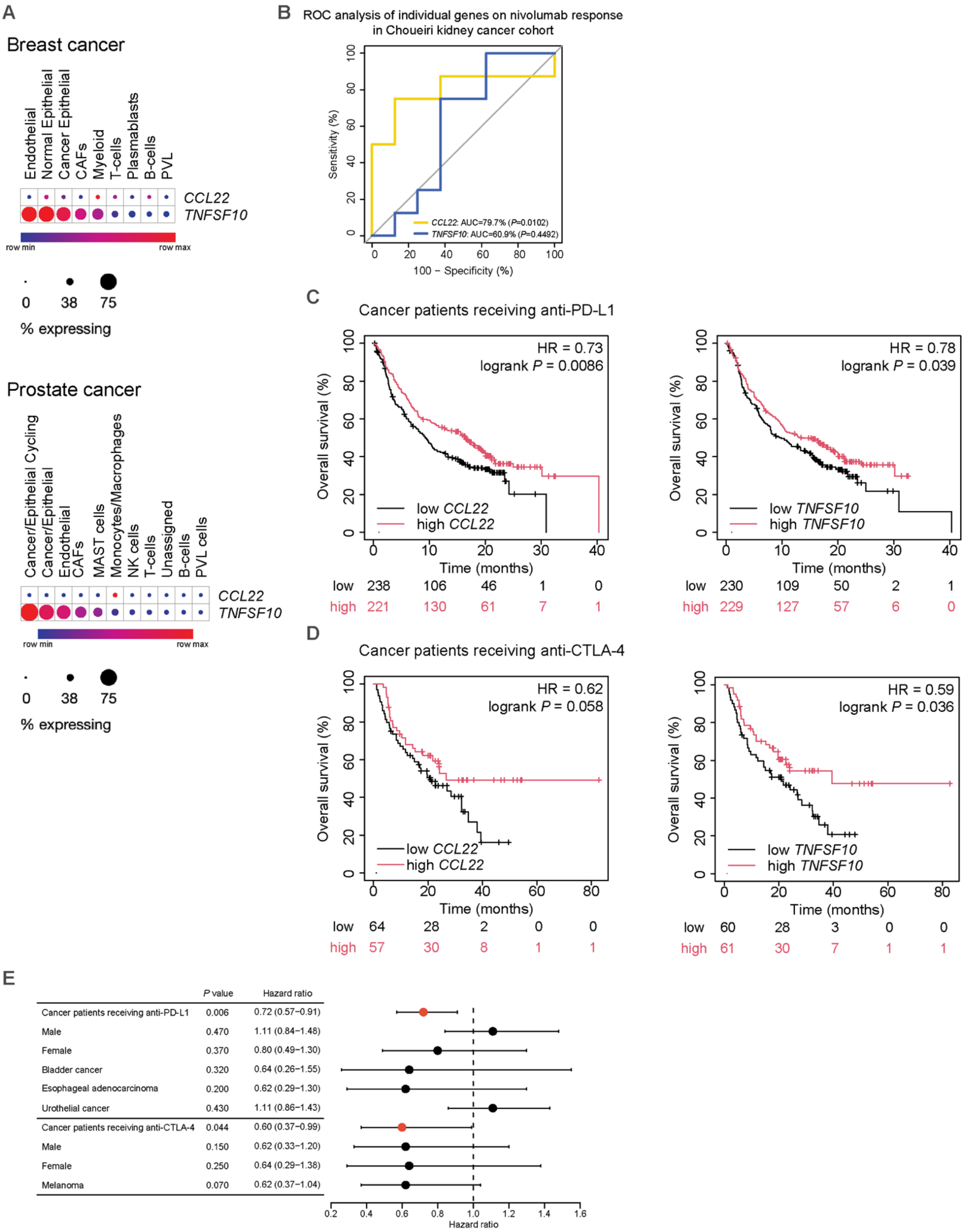
A
Breast cancer
Endothelial
Normal Epithelial
Cancer Epithelial
CAFs
Myeloid
T-cells
Plasmablasts
B-cells
PVL
CCL22
TNFSF10
row min
row max
0
38
75
% expressing
Prostate cancer
Cancer/Epithelial Cycling
Cancer/Epithelial
Endothelial
CAFs
MAST cells
Monocytes/Macrophages
NK cells
T-cells
Unassigned
B-cells
PVL cells
CCL22
TNFSF10
row min
row max
0
38
75
% expressing
B
ROC analysis of individual genes on nivolumab response
in Choueiri kidney cancer cohort
Sensitivity (%)
100 − Specificity (%)
CCL22: AUC=79.7% (P=0.0102)
TNFSF10: AUC=60.9% (P=0.4492)
C
Cancer patients receiving anti-PD-L1
HR = 0.73
logrank P = 0.0086
low CCL22
high CCL22
Overall survival (%)
Time (months)
low 238 106 46 1 0
high 221 130 61 7 1
HR = 0.78
logrank P = 0.039
low TNFSF10
high TNFSF10
Overall survival (%)
Time (months)
low 230 109 50 2 1
high 229 127 57 6 0
D
Cancer patients receiving anti-CTLA-4
HR = 0.62
logrank P = 0.058
low CCL22
high CCL22
Overall survival (%)
Time (months)
low 64 28 2 0 0
high 57 30 8 1 1
HR = 0.59
logrank P = 0.036
low TNFSF10
high TNFSF10
Overall survival (%)
Time (months)
low 60 28 3 0 0
high 61 30 7 1 1
E
P value
Hazard ratio
Cancer patients receiving anti-PD-L1 0.006 0.72 (0.57−0.91)
Male 0.470 1.11 (0.84−1.48)
Female 0.370 0.80 (0.49−1.30)
Bladder cancer 0.320 0.64 (0.26−1.55)
Esophageal adenocarcinoma 0.200 0.62 (0.29−1.30)
Urothelial cancer 0.430 1.11 (0.86−1.43)
Cancer patients receiving anti-CTLA-4 0.044 0.60 (0.37−0.99)
Male 0.150 0.62 (0.33−1.20)
Female 0.250 0.64 (0.29−1.38)
Melanoma 0.070 0.62 (0.37−1.04)
Hazard ratio

Supplementary Figure S5, related to Figure 4. PARP7i and AHRa response biomarker is associated with benefit from ICI therapies.

A. Dot plot showing the expression of *CCL22* and *TNFSF10* across different cell types, as annotated in single-cell RNA sequencing data from human breast and prostate cancers. The scale bar shows the row minimal and row maximal mRNA expression levels and the size of the dot indicates the % of tumors expressing each gene.

B. ROC curves showing the performance of individual biomarker genes *CCL22* and *TNFSF10* in distinguishing response to nivolumab in kidney cancer.

C and D. Kaplan-Meier survival curves showing OS of cancer patients receiving anti-PD-L1 (**C**) or anti-CTLA-4 treatment (**D**). Patients were stratified according to the median expression levels of *CCL22* (*left*) or *TNFSF10* (*right*). Patients with expression above than median were classified as “high” and those below were classified as “low”. Hazard Ratio (HR) and *P* values are included on each plot. The numbers of surviving patients at each time point in the “high” and “low” groups are indicated below each plot.

E. Table and forest plot showing HRs and *P* values of the two-gene biomarker in patients receiving anti-PD-L1 or anti-CTLA-4 therapy (red dots) and subsets of these patients stratified by gender and cancer type (black dots).
