# Supplementary material for: Identification of a Two-Gene Biomarker Correlated with Sensitivity to Combined PARP7 Inhibition and AHR Activation in Cancer Cells
Source: Cancer Res Commun. 2026 Jan 2;6(1):5–16. doi: 10.1158/2767-9764.CRC-25-0173 (PMC12757997; doi:10.1158/2767-9764.CRC-25-0173)
Supplement: Supplementary Figure S6 — , related to Figure 4. PARP7i and AHRa response biomarker offers predictive benefits over some, though not all, markers of immune infiltration. [file crc-25-0173_supplementary_figure_s6_suppsf6.pdf]

A

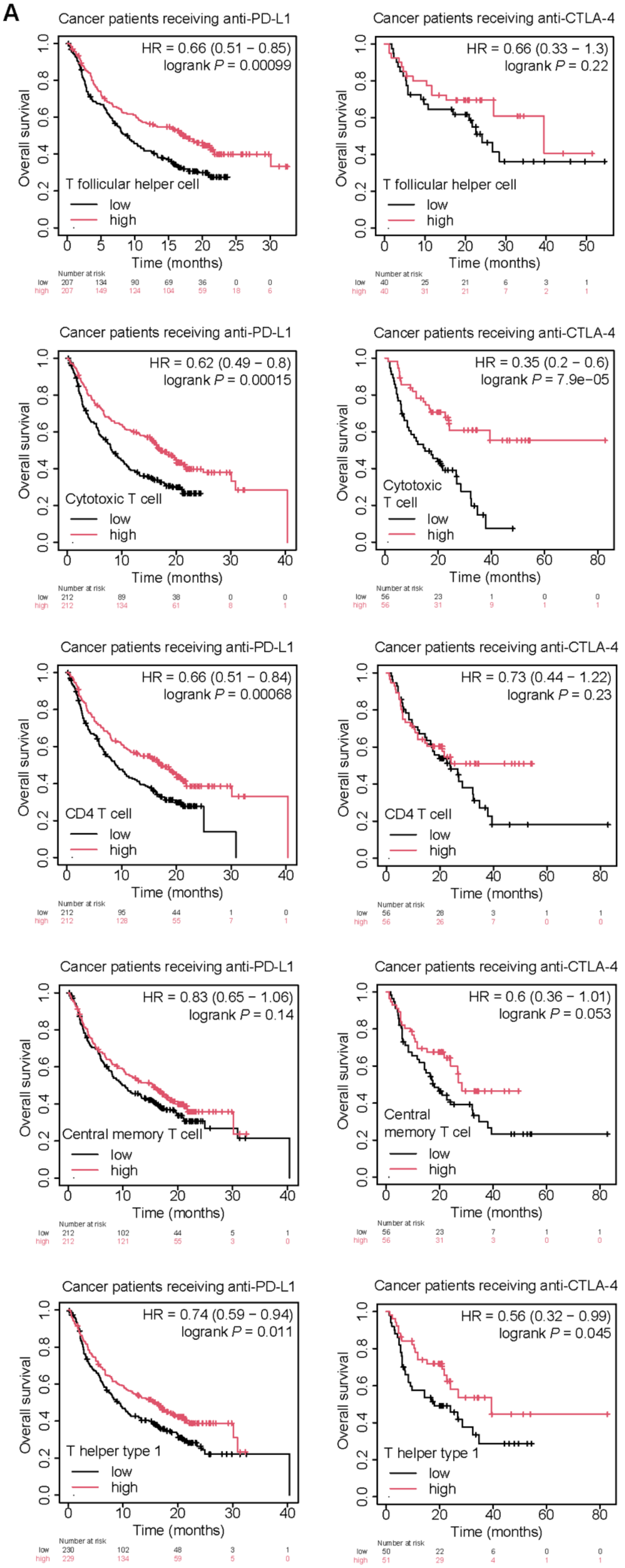

B

Nivolumab response in Choueiri kidney cancer cohort

Gene sets	AUC	*P* values
CIBERSORT.ABS_T_cell_follicular_helper	49.3%	1
EPIC_T_cell_CD4+	31.3%	0.2271
MCP.COUNTER_T_cell	76.6%	0.0831
ESTIMATE_immune_score	79.7%	0.0520

Supplementary Figure S6, related to Figure 4. PARP7i and AHRa response biomarker offers predictive benefits over some, though not all, markers of immune infiltration.

A. Kaplan-Meier survival curves showing the OS of cancer patients receiving anti-PD-L1 (*left*) or anti-CTLA-4 treatment (*right*). Patients were stratified into “high” and “low” groups based on the median value of infiltration scores of each immune cell type. Patients with scores above than median were classified as “high” and those below as “low”. HR and *P* values are included on each plot. The numbers of surviving patients at each time point in the “high” and “low” groups are indicated below each plot.

B. AUCs and *P* values for CIBERSORT.ABS_T_cell_follicular_helper, EPIC_T_cell_CD4+, MCP.COUNTER_T_cell and ESTIMATE_immune_score to predict ICI response in the Choueiri Kidney Cancer Cohort from ClinicalOmicsDB.
